# Inhibitory effects of msFGFR2c on the epithelial-to-mesenchymal transition of AE2 cells in pulmonary fibrosis

**DOI:** 10.1007/s10529-020-02852-x

**Published:** 2020-03-04

**Authors:** Guo Jieming, Chuan Liu, Yin Yang, Shanyi Mo, Xuesong Yang, Ju Wang

**Affiliations:** 1 Institute of Biomedicine, National Engineering Research Center of Genetic Medicine, Jinan University, Guangzhou, 510632 China; 2 Tai Shan College, Shandong University, Jinan, 250000 China; 3 Department of Pediatrics and Neonatology, Institute of Fetal-Preterm Labor Medicine, The First Affiliated Hospital, Jinan University, Guangzhou, 510630 China

**Keywords:** CTGF, FGF-2, msFGFR2c, Pulmonary fibrosis, TGF-β

## Abstract

In interstitial fibrosis, alveolar epithelial type II (AE2) cells fail to repair damaged epithelium. However, whether this dysfunction is related to fibroblast growth factor (FGF) signal pathway and how it affects the fibrotic process remains unclear. In our study, the medium of the human foetal lung fibroblast cell line MRC-5 (Med) can induce epithelial-to-mesenchymal transition (EMT) in AE2 cells, we also found that TGF-β in Med can induce FGF-2 and CTGF expression in AE2 cells. TGF-β or CTGF exposure trigger a FGFR2 subtype b to c transition which can be supressed by siRNA-CTGF. All together, since FGFR2IIIc have the highest affinity with FGF-2 in all of the FGFRs, we indicate the activation of FGF2 signal pathway was induced by TGF-β, which is the key component of Med Here, we also find the inhibitory effect of msFGFR2c (S252W mutant of soluble FGFR2IIIc extracellular domain) on EMT of mouse primary AE2 cells in pulmonary fibrotic process. In a bleomycin-induced mouse pulmonary fibrosis model, msFGFR2c alleviate pulmonary fibrosis and suppress the decrease in pro-SPC levels. Thus, msFGFR2c can inhibit EMT-induced fibrosis of AE2 cells via FGF-2 signal and AE2 cells is suggested to play an important role in the lung fibrotic process.

## Introduction

Pulmonary fibrosis is a respiratory disease characterized by excessive hyperplasia of connective tissues, leading to thickening of the pulmonary alveolar septa, scar formation in lungs, and progressive and serious breathing difficulty (Raghu et al. 2016; Zhang et al. 2012). Idiopathic pulmonary fibrosis (pulmonary fibrosis of unknown origin) (Raghu et al. 2016; Zhang et al. 2012) is a rare disease (Raghu et al. 2006) with poor prognosis and survival time of 2.5–3.5 years after diagnosis (Demedts and Costabel 2002; Society AT 2000). Mammalian alveolar epithelial cells comprise alveolar epithelial type I (AE1) and type II (AE2) cells. AE2 cells synthesize and secrete phospholipid-rich pulmonary surfactants, including SPA, SPB, SPC, and SPD, for preventing alveolar collapse by lowering alveolar surface tension (Zhang et al. 2012; Whitsett et al. 2010), a vital factor for maintenance of alveolar stability and functions. Furthermore, when AE1 cells are damaged, AE2 cells can proliferate and differentiate into AE1 cells to maintain alveolar wall integrity (Tsuji et al. 2009; Miyake et al. 2007). Persistence of pathogen-related chronic pulmonary injury reduced the differentiation of AE2 to AE1 cells and induces the secretion of various profibrogenic cytokines from AE2 cells that promote pulmonary fibrosis (Kim et al. 2006). However, the precise role of this dysfunction in the fibrotic process is unclear.

Transforming growth factor β (TGF-β) is recognized as the most important factor among profibrogenic cytokines (Yang et al. 2014), and it is mainly synthesized and secreted by alveolar macrophages and epithelial cells (Lee et al. 1997). Connective tissue growth factor (CTGF), another profibrogenic cytokine, plays an important role in the formation of pulmonary fibrosis under tissue damage (Robinson et al. 2012); it is expressed at extremely low concentrations under normal conditions and is overexpressed by specific stimuli (e.g., hypoxia or TGF-β) (Yang et al. 2014; Han et al. 2008). Numerous studies have attributed this increased CTGF production to stress fibre production, extracellular matrix protein accumulation, and myofibroblast differentiation (Robinson et al. 2012; Han et al. 2008), and indicate that CTGF is a key mediator contributing to progression of interstitial pulmonary fibrosis.

Previously, we reported a S252W mutant sFGFR2IIIc (msFGFR2) that was bacterially expressed and subsequently renatured (Yu et al. 2012). The binding affinity of FGFR2IIIc for fibroblast growth factor-2 (FGF-2) is the highest as it is the most appropriate receptor for FGF-2 (Powers et al. 2000). We also reported that FGF-2 antibody could almost completely inhibit the α-SMA increase induced by TGF-β, which suggested that FGFR2c might play major role in TGF-beta induced EMT in the AE2 cells since FGFR2c is the most compatible receptor of FGF-2 (Wang et al. 2012). Besides, TGF-β activated the FGF signalling pathway in fibroblasts to promote the fibrotic process, and that wild-type and mutant sFGFR2c (wsFGFR2c and msFGFR2c, respectively) markedly inhibited fibrosis by blocking FGF signal (Robinson et al. 2012; Anderson et al. 1998; Byron and Pollock 2009; Haugsten et al. 2010; Ibrahimi et al. 2004; Wang et al. 2011; Katoh and Katoh 2009; Matsuda et al. 2012). However, the role of FGF signaling in AE2 cell-mediated regulation of pulmonary fibrotic formation is unclear. In this study, we attempted to understand the mechanism on the inhibitory effect of msFGFR2c in this process in vitro and in vivo. Our findings provide insights into the potential application of msFGFR2c in the development of therapies for pulmonary fibrosis.

## Materials and methods

### msFGFR2c expression, renaturation, and purification

We prepared msFGFR2 according to our previously published report (Wang et al. 2011). Briefly, the DNA region from D2 to D3 of the FGFR2IIIc isoform (amino acids 147–366 of hBEK) was amplified from the cDNA, and the S252W mutation was introduced using the splice overlap polymerase chain reaction (PCR) method. The gene fragments were cloned into the pET3c vector and expressed in *Escherichia coli*. Bacteria were collected and lysed, and inclusion bodies were refolded and purified. msFGFR2c with more than 95% purity was harvested using heparin affinity chromatography (GE, Fairfield, CT, USA) (Yu et al. 2012).

### Transfection with siRNA

AE2 cells were transfected with CTGF siRNA or negative control siRNA (GenePharma Co. Ltd. Shanghai, China) using Lipofectamine 2000 transfection reagent (Invitrogen, Carlsbad, CA, USA) in serum-free medium according to the manufacturers’ instructions. After 48 h transfected cells were treated with 4 ng/ml recombinant TGF-β (R&D Systems China Co. Ltd., Shanghai, China) for a further 24 h before proteins were extracted for western blot analysis. The sequence of siRNAs targeting the mouse CTGF gene are shown as follow, sense si-CTGF-1: 5′-GGUCAAGCUGCCCGGGAAATT-3′; antisense si-CTGF-1: 5′-UUUCCCGGGCAGCUUGACCTT-3′; sense si-CTGF-2: 5′-CCCGGGUUACCAAUGACAATT-3′; antisense si-CTGF-2: 5′-UUGUCAUUGGUAACCCGGGTT-3′; sense si-CTGF-3: 5′-CCUGUCAAGUUUGAGCUUUTT-3′; antisense si-CTGF-3: 5′-AAAGCUCAAACUUGACAGGTT-3′; sense si-CTGF-Negative control: 5′-UUCUCCGAACGUGUCACGUTT-3′; antisense si-CTGF-Negative control: 5′ACGUGACACGUUCGGAGAATT-3′.

### Cell lines

Human fetal lung fibroblast cells (MRC-5) and mouse primary AE2 cells were purchased from GuangZhou Jennio Biotech Co. Ltd. (Guangzhou, China), respectively. AE2 cells were cultured in DMEM containing 10% fetal bovine serum (FBS) (Invitrogen, Carlsbad, CA, USA), 1% (v/v) insulin–transferrin–selenium (Sigma, St. Louis, MO, USA), and epidermal growth factor (10 ng/ml; Sigma). MRC-5 cells were cultured in DMEM containing 10% fetal bovine serum (Invitrogen, Carlsbad, CA, USA).

### Experimental animals

C57BL/6 mice were obtained from the Medical Experimental Animal Centre of Guangdong Province (Guangzhou, China). Pulmonary fibrosis was induced in 10-week-old female mice by intratracheal instillation of bleomycin (BLM; Hisun Pharmaceutical Co. Ltd., Zhejiang, China) at 3 mg/kg body weight once in three days. The same amount of saline was intratracheally instilled for the control group. The experimental animals were randomly divided into four groups, namely, those treated with normal saline (control), BLM, or BLM + msFGFR2c. 30 μg msFGFR2c each animal was intraperitoneally injected daily beginning on day 4, and the treated animals were scarified to obtain lung tissue on day 1, 10, 20, and 30 (n = 3). All animal experiments were performed in accordance with the procedures of the Ethical Committee for Animal Experimentation, Jinan University.

### Western blot analysis

Antibodies against the following proteins were used: α-SMA (Millipore, MA, USA), β-actin, CTGF (Santa Cruz Biotechnology, CA, USA), Prosurfactant Protein C (Abcam, Cambridge, UK), N-cadherin, E-cadherin. Horse radish peroxidase (HRP)-conjugated anti-mouse IgG and HRP-conjugated anti-rabbit IgG (Cell Signaling Technology, Danvers, MA, USA) were used as secondary antibodies. All primary antibodies were diluted 2000-fold and secondary antibodies were diluted 10,000-fold in 5% bovine serum albumin (BSA).

### RNA extraction and real-time quantitative PCR analysis

Total RNA was isolated from mouse lung AE2 cells using a TRIzol kit (Invitrogen) according to the manufacturer’s instructions. First-strand cDNA synthesis and SYBR® Green qPCR assays were performed using a PrimeScript™ RT reagent kit (Takara Bio Inc., Shiga, Japan). The specific primers used were: β-actin F: 5′-TGCAGTGGCAAAGTGGAGATT-3′ R: 5′-TTGAAGTCGCAGGAGACAACCT-3′, FGFR1IIIb F: 5′-CGGGAATTAATAGCTCGGATGC-3′ R: 5′-TTGGTGCCGCTCTTCATCTT-3′, FGFR1IIIc F: 5′-GGACTCTCCCATCACTCTGCAT-3′ R: 5′-CCCCTGTGCAATAGATGATGATC-3′, FGFR2IIIb F: 5′-GATAAATAGTTCCAATGCAGAAGTGCT-3′ R: 5′-TGCCCTATATAATTGGAGACCTTACA-3′, FGFR2IIIc F: 5′-GGATATCCTTTCACTCTGCATGGT-3′ R: 5′-TGGAGTAAATGGCTATCTCCAGGTA-3′. Reverse transcription and amplification reactions were performed using the Bio-Rad S1000TM and ABI 7000 (Applied Biosystems, Carlsbad, CA, USA) thermal cyclers, respectively. Gene expression levels were normalized to those of the housekeeping gene β-actin.

### Enzyme-linked immunosorbent assay (ELISA)

MRC-5 and AE2 cells were seeded in 100 mm dishes at a density of 2 × 10^6^/dish and incubated in regular medium overnight; they were then placed in serum-free medium for 24 h. FGF-2, CTGF or TGF-β levels were detected using FGF-2, CTGF or TGF-β ELISA kit (Quantikine™; R&D Systems, Minneapolis, MN, USA) according to the manufacturer’s instructions.

### Hydroxyproline assay

We measured collagen content in the lungs with a conventional hydroxyproline assay using a hydroxyproline kit (Keygen Biochemical Institute, Nanjing, China) according to the manufacturer’s instructions. The experimental results were quantified using a standard curve of known hydroxyproline concentrations.

### Cell migration assay

The Boyden chamber transwell kit from Corning Company was used to evaluate cell migration ability with modifications as described previously (Raghu et al. 2016). Briefly, AE2 cells were harvested and suspended in serum-free endothelial basal medium containing glipizide at the indicated concentrations. Then, the cell suspension (50 µl, 2.5 × 10^4^ cells) was added to the superior chambers. The inferior chambers were filled with media containing 20% FBS and various growth factors, including FGF-2, TGF-β (R&D Systems China Co. Ltd.), and msFGFR2c (produced in our own lab). After incubation at 37 °C for 24 h, cells in the inferior chambers were, stained with 1% crystal violet for 15 min, and counted using a microscope (ECLIPSE Ti-s; Nikon, Tokyo, Japan) at × 40 magnification.

### Photography

Following histochemical staining, images of the desired regions were obtained using a stereo-fluorescence microscope and processed using the Olympus software package Image-ProPlus 7.0. Mouse lungs were treated by triformol, then treated with OCT mounting medium before sectioned at 10 µm using a cryostat microtome (Leica CM1900; Leica, Wetzlar, Germany). Images were obtained using an Olympus IX51 epi-fluorescent microscope (at × 200 and × 400) (Olympus, Tokyo, Japan) and analysed using the CW4000 FISH Olympus software.

### Histology

The mouse lungs were fixed in 4% paraformaldehyde at 4 °C for 24 h. The lung specimens were then dehydrated, cleared in xylene, embedded in paraffin wax, and serially sectioned at 5 µm using a rotary microtome (RM2126RT; Leica). The sections were stained hematoxylin and eosin (H&E). Airspace volume density was measured using minimum five random images from three samples per group and time point. Masson’s staining was used to detect fibrosis in lung sections.

### Data analysis

All data analyses were performed using GraphPad Prism 5 (GraphPad Software, La Jolla, CA, USA). The results are presented as mean values ± standard deviation. All data were analyzed using analysis of variance (ANOVA) or Student’s *t*-test to establish differences between the experimental and control groups. Each experiment was repeated three times. *p* Values less than 0.05 were considered significant.

## Results

### msFGFR2c restricted FGF-2-induced or TGF-β-induced AE2 cell migration in vitro

**Fig. 1:**
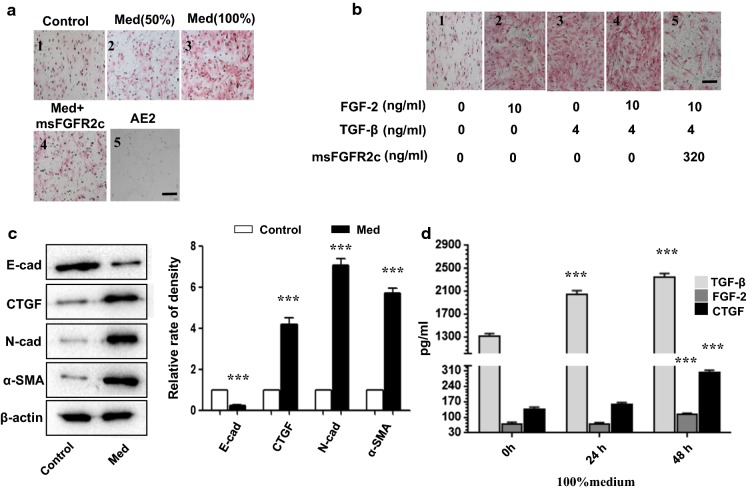
Determination of alveolar epithelial type II (AE2) cell migration using a transwell assay following exposure to Med (culture medium collected after 48 h of incubation with MRC-5 cells) and TGF-β in combination with msFGFR2c. **a** Representative crystal violet-stained transwell images for control (**a1**), 50% Med-treated (**a2**), 100% Med-treated (**a3**), 100% Med + 320 ng/ml-treated msFGFR2c (**a4**)-treated AE2 cells, (**a5**) on behalf of the AE2 cells under bright-field microscope. **b** Crystal violet-stained Transwell images of AE2 cells pre-treated for 24 h with buffer, 10 ng/ml FGF-2, 4 ng/ml TGF-β, 10 ng/ml FGF-2 + 4 ng/ml TGF-β, 10 ng/ml FGF-2 + 4 ng/ml TGF-β + 320 ng/ml msFGFR2c. **c** Western blot data showing the protein levels of E-cadherin, CTGF, N-cadherin, and α-SMA in AE2 cells from the control and Med-treated groups. The bar chart shows the ratios of band densities from western blot data. **d** The bar charts show the levels of TGF-β, FGF-2 and CTGF in the MRC-5 cell culture medium at 0, 24, and 48 h. Scale bars = 100 µm in **a** and **d**. *Indicates comparison with the control (***p < 0.001)

To investigate whether msFGFR2c affects AE2 cell migration, a transwell assay was performed by 24 h incubation of AE2 cells with the medium of MRC-5 cells after 48 h culture (referred to as Med) and various growth factors (Fig. 1a, b). Not only 50% (Fig. 1a2) and 100% (Fig. 1a3) Med promoted AE2 cell migration in a dose-dependent manner, but also TGF-β and FGF-2 did. However, addition of 320 ng/mL msFGFR2c (Fig. 1a4) to the culture medium dramatically decreased the number of cells that passed through the wells in Med and TGF-β + FGF-2 groups, suggesting that TGF-β was important in this prosess, and FGF signal block could neutralize the pro-migratory effects of TGF-β and FGF-2. The assay (Fig. 1b) also shows that TGF-β and FGF-2 can promote AE2 cell migration seperately, and have stronger effect when they are combined. Meanwhile, their effect can both be negated by msFGFR2c, hinted that they all function through FGFR2c. Western blot results showed that CTGF, N-cadherin, and α-SMA, all pro-fibrotic factors, in AE2 cells in the presence of Med (Fig. 1c) were all up-regulated, whereas E-cadherin, the epithelial cell marker, was down-regulated (Fig. 1c).

Furthermore, in order to understand the location of a cytokine in the signaling pathway, FGF-2, TGF-β and CTGF levels in the MRC-5 cell culture medium were measured by ELISA at 24 h and 48 h. Nanogram level of TGF-β was secreted, which started increasing significantly from the start in a time-dependent manner (Fig. 1d), suggested that TGF-β might play a major role. CTGF levels started increasing from the beginning of the 48 h incubation period (Fig. 1d), meanwhile FGF-2 level increased later.

All these results indicated that TGF-β, FGF2 and CTGF secreted by MRC-5 cells might play important roles in inducing epithelial-to-mesenchymal transition (EMT) in the AE2 cells.

### TGF-β switched a shift in the FGFR subtypes through CTGF and promoted FGF-2 secretion in AE2 cells, thereby its induction of fibrosis could be blocked by msFGFR2c

**Fig. 2:**
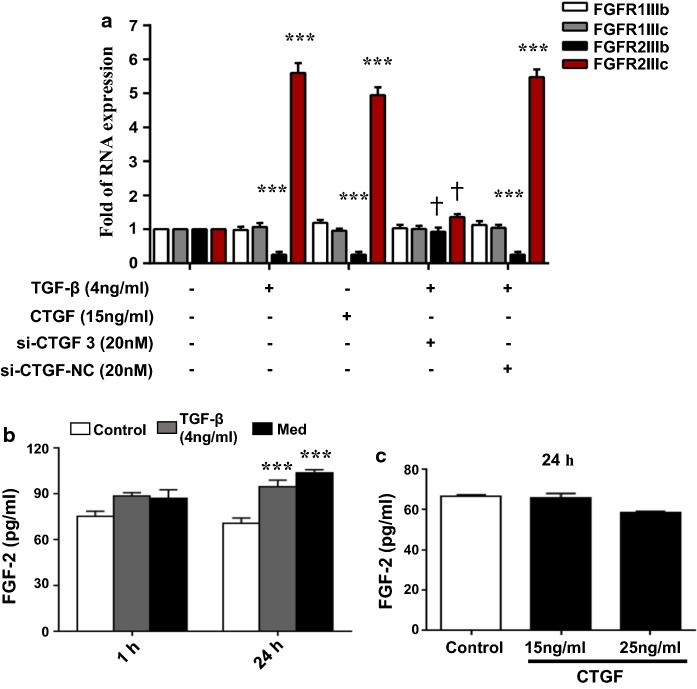
The levels of FGF receptor subtypes in AE2 cells in the presence of CTGF, and levels of FGF-2 in AE2 cells following stimulation with either TGF-β or Med. **a** Bar chart summarising the RT-PCR expression data for FGF receptor subtypes (FGF1IIIb, FGF1IIIc, FGF2IIIb, and FGF2IIIc) in AE2 cells following stimulation with 4 ng/ml TGF-β, 20 nM siRNA-CTGF or 15 ng/ml CTGF for 24 h. **b** ELISA results showing FGF-2 levels in AE2 cells after 1 h and 24 h stimulation with either 4 ng/ml TGF-β or 100% Med. **c** ELISA results showing FGF-2 levels in AE2 cells at 24 h after stimulation with either 15 ng/ml CTGF or 25 ng/ml CTGF. *Indicates comparison with the control; † indicates comparison with the induced groups (***^,†^p < 0.001)

The levels of various FGFR subtypes, including FGFR1IIIb, FGFR1IIIc, FGFR2IIIb, and FGFR2IIIc in AE2 cells were determined by real-time PCR after the addition of 4 ng/ml TGF-β or 15 ng/ml CTGF for 24 h (Fig. 2a). According to previous research, CTGF and TGF-β both function through FGF signal pathway, which means they need the FGFR2IIIb to FGFR2IIIc subtype switch to function. So we mainly focus on the subtype switch. Compared to the control, there were no obvious alterations in the levels of FGFR1 subtypes induced by TGF-β and CTGF. However, FGFR2b expression was down-regulated and FGFR2c expression was up-regulated by TGF-β and CTGF treatment dramatically (Fig. 2a). When siRNA-CTGF was added, it revers the promoting effect of the TGF-βon the switching of FGFR2b to FGFR2c. This result suggested that TGF-β promoted a shift of the FGFR2 subtype expression from FGFR2b to FGFR2c through CTGF signal pathway.

Furthermore, FGF-2 secretion in the AE2 cell culture medium detected by ELISA (Fig. 2b) increased significantly at 24 h, but not at 1 h, following stimulation with 4 ng/ml TGF-β or 100% Med (Fig. 2b). Meanwhile, FGF-2 secretion did not change distinctly after the addition of 15 or 25 ng/ml CTGF in AE2 cells (Fig. 2c) but the inverse was true (Fig. 2b). Therefore, we speculated that the elevation of FGFR2c level induced by TGF-β and CTGF made the enhancement of FGF-2 response in AE2 cells, as FGFR2c have the highest affinity with FGF-2 (Mohammadi 2005). Summing-up, FGF-2 signal became dramatically strong and functioned as promoting factor in lung fibrossis.

### msFGFR2c application alleviated BLM-induced mouse pulmonary fibrosis

**Fig. 3:**
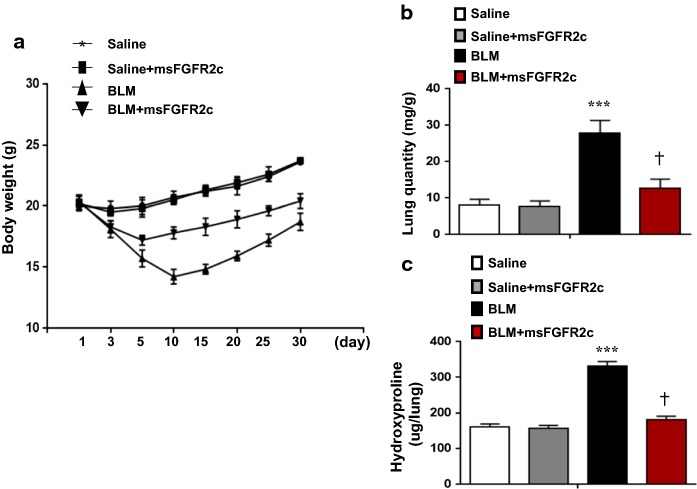
Effect of msFGFR2c application on bleomycin-induced pulmonary fibrosis. A mouse model for pulmonary fibrosis was established by intratracheal bleomycin (BLM) instillation. **a** The mouse body weights were measured within 1 month after exposure to BLM, msFGFR2c, or/and saline(as control). **b** The pulmonary quantity (pulmonary weight/body weight) was determined for different groups. **c** The hydroxyproline contents (µg/lung) in mouse lungs were determined for different groups. *Indicates comparison with the control. ^†^Indicates comparison with the induced groups (***^,†^p < 0.001)

To confirm the anti-fibrotic effect of msFGFR2c in vivo, we established a mouse pulmonary fibrosis model by intratracheal BLM instillation in 10-week-old female mice. In our study, we observed that msFGFR2c treatment significantly reversed the BLM-induced reduction of mouse body weight (Fig. 3a) and increased pulmonary quantity (Fig. 3b) and pulmonary hydroxyproline content (Fig. 3c).

**Fig. 4:**
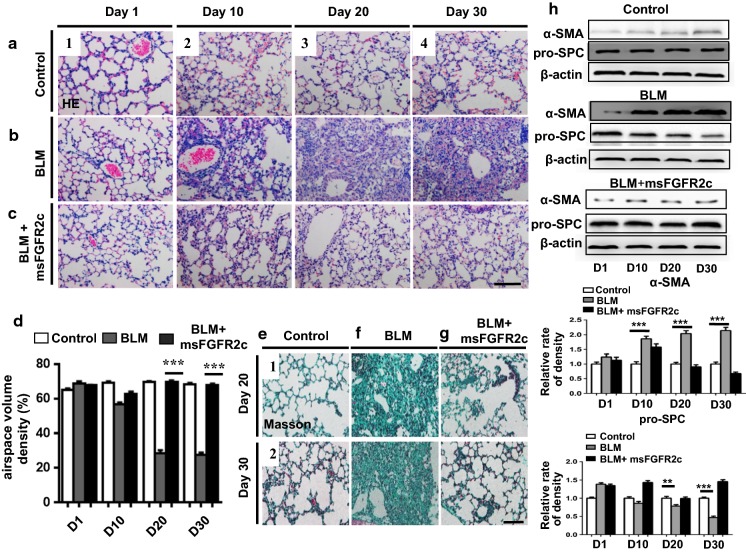
Mouse pulmonary histology was characterised by haematoxylin and eosin (H&E) or Masson’s trichrome stain in mice treated with bleomycin (BLM) and BLM + msFGFR2c. **a**–**c** Representative H&E-stained lung transverse section images obtained at days 1 (**a1**), 10 (**a2**), 20 (**a3**), and 30 (**a4**) for mice in the control group; days 1 (**b1**), 10 (**b2**), 20 (**b3**), and 30 (**b4**) for mice in the BLM-treated group; days 1 (**c1**), 10 (**c2**), 20 (**c3**), and 30 (**c4**), for mice in the BLM + msFGFR2c-treated group. **d** Bar chart shows the comparison of airspace volume densities among the control, BLM, and BLM + msFGFR2c groups. **e**–**g** Representative Masson-stained lung transverse section images obtained at days 20 (**e1**) and 30 (**e2**) for mice in the control group, days 20 (**f1**) and 30 (**f2**) for mice in the BLM-treated group, and days 20 (**g1**) and 30 (**g2**) for mice in the BLM + msFGFR2c-treated group. Scale bars = 100 µm in (**a**–**c**) and (**e**–**g**). **h** Western blot data showing the protein levels of pro-SPC and α-SMA on the corresponding treatment days for the control, BLM-treated, and BLM + msFGFR2c-treated groups. *Indicates comparison with the control (***p < 0.001)

Next, H&E-stained paraffin sections revealed that BLM treatment induced pulmonary fibrosis beginning from day 10, which showed gradual aggravation (Fig. 4b) compared to the control (Fig. 4a). BLM-induced pulmonary fibrosis was alleviated by msFGFR2c addition (Fig. 4b–c). These morphological observations were supported by the measurement of pulmonary airspace volumes (Fig. 4d). Similarly, we observed a reversal of BLM-induced pulmonary fibrosis upon msFGFR2c treatment in Masson-stained lung sections on days 20 and 30 (Fig. 4e–g).

Furthermore, since pro-SPC is the marker of AE2 cells (Almeida et al. 2013) and high expression of α-SMA is the hall-marker of myofibroblasts (Hinz et al. 2003), (the key effector cells in the development of fibrosis), the quantitative expression of pro-SPC and α-SMA at the protein level in mouse lungs was used as markers and was determined by western blotting after BLM or BLM + msFGFR2c administration on different days. After treatment with BLM alone, α-SMA expression increased in a time-dependent manner compared to the levels in the corresponding controls, whereas additional treatment with msFGFR2c on day 30 reversed these changes, suggesting that msFGFR2c reverse the fibertic process induced by BLM (Fig. 4h). Pro-SPC levels decreased in the presence of BLM after a combined treatment with msFGFR2c on day 30 and gradually returned to normal levels, suggesting that mdFGFR2c prevent the decrease of AE2 cells (Fig. 4h). AE2 cells decreasing is a key feature of the fibrotic process and results above suggests that msFGFR2c could reverse this trend, thus preventing the EMT from being activated.

## Discussion

The regenerative AE2 cells fail to repopulate the denuded epithelial basal lamina during fibrosis. Though AE2 cells play a key role in lung fibrosis and epithelial regeneration (Knudsen et al. 2017), the precise role of AE2 cells in the fibrotic process and the function of FGF signalling in AE2 cells remain unknown.

Owing to their crucial role in pulmonary fibrosis (Corti et al. 1996; Ehrhardt et al. 2005; Fujino et al. 2011; Liu et al. 2016), mouse primary AE2 cells were examined in this study. Here, we found that the addition of fibroblast medium of MRC-5 cells increased N-cadherin and α-SMA levels and decreased E-cadherin levels in AE2 cells (Fig. 1c). Since MRC-5 cell line comes from human being, primary AE2 cells come from mice, we could see TGF-β, FGF-2 and CTGF from MRC-5 cells take their effects on the primary mouse AE2 cells because of the high homology of these cytokines in human and mouse. Though these experiments should be done more strictly in cells from the same species, we just used mouse primary AE2 cells since human primary AE2 cells was very difficult to be obtained.

Furthermore, msFGFR2c inhibited the pro-fibrotic effect of TGF-β, FGF-2, or CTGF, suggesting that the role of a shift in FGFR subtypes from FGFR2b to FGFR2c and FGF-2 increase were important for fibrosis (Fig. 3). Thus, FGF-2 signalling pathway activation in AE2 cells might be the key point in the pulmonary fibrotic process.

In the mouse BLM-induced lung fibrotic model, we found msFGFR2c markedly inhibited the fibrotic process. In the msFGFR2c treatment group, the marker of AE2 cells, pro-SPC, sustained its expressional level, but in the BLM-induced fibrotic group, pro-SPC was down-regulated dramatically in the whole lung-tissue WB assay. These results also suggested the importance of AE2 cells in the repairing of BLM-induced lung wound. Blocking FGF2 signal pathway could inhibit the EMT transition of AE2 cells and further retard lung fibrotic process.

Summing up, we found that TGF-β promoted FGF-2 expression, triggered FGF receptor subtype b to c transition via CTGF, and promoted EMT in AE2 cells. Therefore, msFGFR2c could inhibit the promoting effects of TGF-β, CTGF, and FGF-2 during lung fibrosis and has the potent clinical value for the treatment of pulmonary fibrosis.
